# Appropriate household water treatment methods in Ethiopia: household use and associated factors based on 2005, 2011, and 2016 EDHS data

**DOI:** 10.1186/s12199-018-0737-9

**Published:** 2018-09-27

**Authors:** Abraham Geremew, Bezatu Mengistie, Jonathan Mellor, Daniele Susan Lantagne, Esayas Alemayehu, Geremew Sahilu

**Affiliations:** 1 Ethiopian Institute of Water Resources, Addis Ababa University, Addis Ababa, Ethiopia; 2 Department of Environmental Health Sciences, College of Health and Medical Sciences, Haramaya University, Dire Dawa, Ethiopia; 3 Department of Civil and Environmental Engineering, University of Connecticut, Mansfield, USA; 4 Department of Civil and Environmental Engineering, Tufts University, Medford, USA; 5 Department of Civil and Environmental Engineering, Institute of Technology, Jimma University, Jimma, Ethiopia

**Keywords:** Education of household head, Household use, Water treatment, Wealth status

## Abstract

**Background:**

Diarrheal disease attributable to water and sanitation can be prevented using point-of-use water treatment. In Ethiopia, a small number of households treat water at point-of-use with appropriate methods. However, evidence on factors associated with household use of these treatment methods is scarce. Therefore, this study is intended to explore the household use of appropriate point-of-use water treatment and associated factors in Ethiopia.

**Methods:**

The data of 2005, 2011, and 2016 Ethiopian demographic and health surveys were used for analysis. Households reportedly treating water with bleach, boiling, filtration, and solar disinfection in each survey are considered as treating with appropriate treatment methods. Household water treatment with these treatment methods and factors associated was assessed using bivariate and multivariable regression. In addition, a region level difference in the treatment use was assessed by using multilevel modeling.

**Results:**

The number of households that reported treating water with appropriate water treatment methods was 3.0%, 8.2%, and 6.5% respectively in 2005, 2011, and 2016. Household heads with higher education had 5.99 (95% CI = 3.48, 10.33), 3.61 (95% CI = 2.56, 5.07), and 3.43 (95% CI = 2.19, 6.37) times higher odds of using the treatment methods respectively in 2005, 2011, and 2016 compared to household heads who had no education. There was a significantly high number of households that used appropriate water treatment methods in 2011 (AOR = 2.78, 95% CI = 2.16, 3.57) and 2016 (AOR = 2.18, 95% CI = 1.64, 3.89) compared to 2005 data. In pooled data analysis, the reported use of the treatment methods is associated with household head education, residency, drinking water sources, and owning radio and television. From a multilevel modeling, within-region variation is higher than between-region variations in the use of treatment methods in each survey.

**Conclusions:**

Below 10% of households reportedly treating water at point-of-use in each survey attributable to different factors. Designing intervention strategies for wide-scale use of treatment methods at the country level is fundamental.

## Background

Diarrhea is among the leading cause of mortality and morbidity in developing countries [1]. The recent estimate shows that it is the fourth leading cause of death globally in children under 5 years, with 38% of all deaths [2]. Death from diarrheal disease in developing countries is mostly attributable to inadequate water, sanitation, and hygiene [3]. Various preceding systematic review findings suggest that point-of-use water treatment is one of the methods that can reduce this burden [4–6]. However, household adoption and sustained use are challenges [6]. A systematic review of household adoption and use of water, sanitation, and hygiene technologies categorizes the factors into three broad categories: psychosocial, contextual, and technology-related factors [7].

In Ethiopia, there are different point-of-use water treatment options being practiced of which boiling, adding bleach, filtration, and solar disinfection are listed as appropriate point-of-use water treatment methods [8, 9]. There has been an effort to improve the coverage of these water treatment options in the country [10]. The Health Transformation Plan of the country (2016–2020) shows that it is targeted to reach 35% coverage in the household use of water treatment methods and safe storage practices by 2020 [11]. However, the use of point-of-use water treatment methods is still low in Ethiopia [8, 9]. Moreover, there is reporting on the number of households using these water treatments using the demographic and health surveys conducted every 5 years in the country. But advanced analysis of the data to show factors associated with households use of these treatment methods and the region level differences in the use of treatment has not yet been conducted for evidence-based intervention. Therefore, the current study is intended to assess the household use of appropriate point-of-use water treatment methods and associated factors, and region level differences in the treatment use using the three demographic and health surveys. The findings from this study can be used by concerned bodies to design a strategy for a wide-scale use of treatment methods for the ultimate health gain of reducing the burden of diarrheal disease in the country. In addition, the finding can be a basis for further study.

In the current analysis, it is hypothesized that the reported use of appropriate treatment methods is high in the households of educated household heads, high wealth quintiles, urban dwelling, owned a radio, owned a television, dependent on unimproved water sources, having under 5-year-old children, and male household head.

## Methods

### Date source

The Ethiopian Demographic and Health Survey (EDHS) conducted in 2005, 2011, and 2016 were used as a data source for analysis. The surveys were conducted based on a nationally representative sample households that provide estimates at the national and regional levels. Women of child-bearing age 15–49 and all men aged 15–59 in randomly selected households across the country were the target groups for the surveys [8, 9, 12]. This study considered all households in the country in each survey as a targeted population as it was intended to generalize the point-of-use water treatment practices at the national level. Data collection was taken place by interviewing respondents from the selected households [8, 9, 12]. The Federal Ministry of Health supported the survey implemented by the Central Statistical Agency with technical assistance from the ICF International through its MEASURE DHS project. MEASURE DHS is a 5-year project to assist institutions in collecting and analyzing data needed to plan, monitor, and evaluate population, health, and nutrition programs [13].

### Sample size and sampling technique

The sample in each EDHS was designed to provide population and health indicators at the national and regional levels. The sample design allowed for specific indicators to be calculated for each of Ethiopia’s 11 administrative regions: 9 regional states (Tigray, Afar, Amhara, Oromiya, Somali, Benishangul-Gumuz, Southern Nations Nationalities and Peoples (SNNP), Gambela, and Harari) and 2 city administrations (Addis Ababa and Dire Dawa). Accordingly, 14,500 households from 540 clusters in 2005 EDHS; 17,817 households from 624 clusters in 2011 EDHS, and 16,650 households from 645 clusters in 2016 EDHS were selected for the surveys. In each survey, each of the regions was stratified into urban and rural areas, and samples of enumeration areas (EAs) were selected independently in each stratum. The samples were selected using a stratified, two-stage cluster design. First, clusters were selected from the list of enumeration areas from the population and housing census sample frame (1994 census for 2005 EDHS and 2007 census for 2011 and 2016 EDHS). Second, households were selected after listing of households in all of the selected EAs for sampling frame. The detail of the sampling technique is found elsewhere [8, 9, 12].

### Study variables

Households reported using appropriate water treatment methods were considered for analysis as an outcome of an interest. According to 2011 and 2016 EDHS reports, adding bleach, boiling, filtration, and solar disinfection (SODIS) were taken as using appropriate water treatment methods. Therefore, households’ reported use of boiling, adding bleach, filtering, or solar disinfecting took a binary form such that the use was considered a yes (1 = if the household had used either of the methods during each survey) and no otherwise (0 = if the household had used neither during each survey). The predictor variables are residency of households (categorized into urban and rural), household wealth status categorized into five quintiles (poorest, poorer, middle, higher, and highest), presence of under 5-year-old child in the household, education status of household head, owning television and radio, type of water sources (improved versus unimproved), sex of household head, age of household head, and intermittent water supply.

### Data analysis

The “svy” command in Stata was used to weight the survey data for the adjustment of cluster sampling design. The weighted data were analyzed using descriptive (frequency and proportion), bivariate, and multivariable regression analysis. Bivariate regression was applied to determine unadjusted effects of each of the variables on household use of the treatment methods during each survey. Then, we subsequently included the variables with a *p* value < 0.25 for multivariable regression to assess the independent effect after controlling other variables [14]. In addition, a two-level multilevel modeling was employed to determine whether the effect of identified predictors vary due to regions. The two-level multilevel logistic regression that considers a collection of groups (regions) and within-group (random households selected within regions) is used for analysis because data from a random sample of level 1 households were collected from level 2 regions. For all analysis, the significant association of predictor variables was considered at *p* value < 0.05. All statistical analyses were conducted using Stata version 14.0 (Stata Corp, College Station, TX, USA). Multi-collinearity diagnostics were conducted to exclude the variable/s with the variance inflation factor (VIF) of greater than 10 from multivariable regression.

## Results

### Household characteristics and drinking water sources

Data about appropriate water treatment and characteristics attributable to it were analyzed using 13,711, 16,672, and 16,650 households respectively for 2005, 2011, and 2016 EDHS. Table 1 shows the household characteristics of the three surveys. Of total household heads, about 68% in 2005, 57% in 2011, and 55% in 2016 had no formal education. A small number of households (4.9%) owned a television in the 2005 survey, and the number increased to 13.8% in 2016 survey. More than 70% of adult women were responsible to fetch water in each of the surveys. There was an increasing trend in the number of households using improved water sources in three surveys. The number of households accessed the water on the premises was 8.4% in 2005 and increased to 20.1% in the 2016 survey. The number of households that accessed drinking water supplies within 30 min in 2011 and 2016 surveys was almost the same.

**Table 1:** The characteristics of the households in the DHS of 2005, 2011, and 2016, Ethiopia

Household characteristics		EDHS 2005, *n* (%)	EDHS 2011, *n* (%)	EDHS 2016, *n* (%)
Education status of head	No education	9236 (67.5)	9526 (57.2)	9083 (54.7)
	Primary	2943 (21.5)	5325 (32.0)	5028 (30.3)
	Secondary	1214 (8.9)	896 (5.4)	1324 (8.0)
	Higher	287 (2.1)	910 (5.5)	1168 (7.0)
Wealth status	Poorest	2754 (20.1)	3208 (19.2)	3202 (19.2)
	Poorer	2838 (20.7)	3219 (19.3)	3203 (19.2)
	Middle	2668 (19.5)	3091 (18.5)	3121 (18.8)
	Higher	2529 (18.4)	3067 (18.4)	3084 (18.5)
	Highest	2923 (21.3)	4102 (24.6)	4040 (34.3)
Sex of household head	Male	10,594(77.3)	12,335 (73.9)	12,426 (74.6)
	Female	3117 (22.7)	4352 (26.1)	4224 (25.4)
Person fetching water	Adult woman	10,209 (81.4)	10,404 (71.3)	9612 (72.2)
	Adult man	806 (6.4)	1277 (8.8)	1193 (9.0)
	Female below 15 years old	1113 (8.9)	2065 (14.1)	1726 (13.0)
	Male below 15 years old	329 (2.6)	701 (4.8)	576 (4.3)
	Other	85 (0.7)	154 (1.0)	204 (1.5)
Radio ownership	No	9090 (66.3)	9917 (59.4)	11,952 (71.8)
	Yes	4617 (33.7)	6767 (40.6)	4698 (28.2)
Television ownership	No	13,039 (95.1)	14,947 (89.6)	14,354 (86.2)
	Yes	668 (4.9)	1730 (10.4)	2296 (13.8)
Residency	Rural	11,741 (85.6)	12,915 (77.4)	13,266 (79.7)
	Urban	1970 (14.4)	3772 (22.6)	3384 (20.3)
Water sources	Unimproved sources	8185 (59.7)	7730 (46.3)	5857 (35.2)
	Improved sources	5526 (40.3)	8957 (53.7)	10,793 (64.8)
Distance of water sources	Water on premises	1147 (8.4)	2075 (12.4)	3339 (20.1)
	< 30 min (round trip)	6076 (44.3)	5774 (34.6)	5871 (35.3)
	≥ 30 min (round trip)	6489 (47.3)	8838 (53.0)	7440 (44.7)

Households with unknown response to the water distance were categorized into > 30 min in all three surveys; therefore, column total may not be equal to national reports

### Household reported the use of water treatment methods

The number of households treating their water prior to drinking with any treatment options was 8.0% in 2005, 10.2% in 2011, and 9.4% in 2016 (Table 2). In 2005 EDHS data, a total of 3.0% of households had used one of the appropriate treatment methods. Explicitly, 2.4% of households had reportedly used boiling, 0.2% of households had reportedly used bleach, and 0.3% of households had reportedly used a filter as point-of-use water treatment methods. In 2011, the number of households that reportedly used boiling, bleach, and filter as household water treatment methods was 2.7%, 5.8%, and 0.2%, respectively. In this survey, there was no household that reportedly used SODIS. The total number of households that reportedly used the appropriate household water treatment methods in 2011 was 8.2%. In 2016, 6.5% of households had reportedly used either of the listed treatment methods to treat their drinking water prior to drinking. Specifically, 2.2%, 3.4%, 1.1%, and 0.1% was the number of households that reportedly used boiling, bleach, filter, and SODIS, respectively, as a household water treatment method. In general, appropriate water treatment use is least in the 2005 survey and highest in 2011 survey.

**Table 2:** Type of water treatment methods used by households in DHS of 2005, 2011, and 2016, Ethiopia

Treatment type		Household use in survey year		
		2005, weighted frequency (%)	2011, weighted frequency (%)	2016, weighted frequency (%)
All treatment methods	Boiling	335 (2.4)	450 (2.7)	364 (2.2)
	Bleach	32 (0.2)	973 (5.8)	567 (3.4)
	Cloth straining	626 (4.6)	206 (1.2)	331 (2.0)
	Filtration	47 (0.3)	40 (0.2)	182 (1.1)
	SODIS	0	0	11 (0.1)
	Let it stand and settle	21 (0.2)	21 (0.1)	59 (0.4)
	Other	42 (0.3)	35 (0.2)	28 (0.2)
	Total	1103 (8.0)	1725 (10.2)	1542 (9.4)
Appropriate treatment methods (boiling, adding leach, filtration, or SODIS)		409 (3.0)	1368 (8.2)	1081 (6.5)

SODIS = 0 is due to the weighting of data; number of households using appropriate treatment methods is obtained by weighting the sum of the number of households reportedly using boiling, bleach, filter, and SODIS

### Result of bivariate and multivariable analysis

Table 3 indicates the bivariate regression of the household use of appropriate point-of-use water treatment methods in the three surveys. In the bivariate regression analysis, the socio-demographic and economic characteristics of households were associated with the use of appropriate point-of-use water treatment methods. The odds of using appropriate point-of-use water treatment methods in urban area are 1.51 (95% CI = 1.03, 2.22) times higher in 2005, 1.80 (95% CI = 1.32, 2.45) times higher in 2011, and 1.58 (95% CI = 1.17, 2.13) times higher in 2016 compared to rural area. Household head with higher education had the odds of 7.42 (95% CI = 4.78, 11.52) times higher in 2005, 6.65 (95% CI = 4.61, 9.58) times higher in 2011, and 3.27 (95% CI = 2.29, 4.67) times higher in the reported use of appropriate water treatment methods compared to house head who had no education. The odds of reported use of appropriate treatment methods among households found in the highest wealth quintile in 2005, 2011, and 2016 were 2.77 (95% CI = 1.39, 5.54), 5.02 (95% CI = 3.19, 7.90), and 1.83 (95% CI = 1.06, 3.18) times higher, respectively, than households found in poorest wealth quintile.

**Table 3:** Bivariate regression result on factors associated with the household use of appropriate water treatment methods in DHS of 2005, 2011, and 2016, Ethiopia

Household characteristics		2005 EDHS			2011 EDHS			2016 EDHS		
		User	Non-user	COR (95% CI)	User	Non-user	COR (95% CI)	User	Non-user	COR (95% CI)
Residency	Rural	327	11,414	1.0	914	12,002	1.0	724	12,542	1.0
	Urban	82	1888	1.51 (1.03, 2.22)*	454	3318	1.80 (1.32, 2.45)*	357	3027	1.58 (1.17, 2.13)*
Education of head	No education	177	9059	1.0	486	9040	1.0	377	8706	1.0
	Primary	134	2809	2.43 (1.80, 3.28)*	493	4832	1.89 (1.52, 2.36)*	380	4647	1.66 (1.32, 2.09)*
	Secondary	60	1154	2.66 (1.76, 4.03)*	143	753	3.52 (2.47, 5.02)*	136	1188	1.96 (1.40, 2.75)*
	Higher	36	250	7.42 (4.78, 11.52)*	240	670	6.65 (4.61, 9.58) *	187	981	3.27 (2.29, 4.67)*
Wealth status	Poorest	50	2704	1.0	111	3097	1.0	165	3037	1.0
	Poorer	65	2773	1.26 (0.72, 2.19)	151	3069	1.37 (0.89, 2.11)	128	3076	0.84 (0.53, 1.35)
	Middle	50	2617	1.04 (0.55, 2.00)	177	2914	1.69 (1.09, 2.61) *	150	2971	1.07 (0.60, 1.92)
	Higher	102	2427	2.28 (1.24, 4.19)*	303	2764	3.06 (1.99, 4.71)*	201	2882	1.34 (0.75, 2.41)
	Highest	142	2781	2.77 (1.39, 5.54)*	626	3476	5.02 (3.19, 7.90)*	436	3603	1.83 (1.06, 3.18)*
Water sources	Improved	156	5370	1.0	797	8159	1.0	384	10,096	1.0
	Unimproved	253	7931	1.10 (0.79, 1.53)	571	7160	0.82 (0.60, 1.12)	697	5473	1.12 (1.00, 1.25)
Had radio	No	188	8901	1.0	522	9395	1.0	658	11,293	1.0
	Yes	221	4396	2.38 (1.71, 3.31)*	846	5922	2.57 (2.06, 3.20)*	423	4276	1.52 (1.26, 1.84)*
Had television	No	368	12,671	1.0	995	13,952	1.0	762	13,592	1.0
	Yes	41	627	2.26 (1.57, 3.26)*	371	1359	3.83 (2.78, 5.26)*	319	1977	2.21 (1.63, 3.00)*
Sex of house head	Female	103	3014	1.0	980	11,355	1.0	776	11,650	1.0
	Male	306	10,288	1.15 (0.82, 1.62)	388	3965	1.13 (0.93, 1.38)	305	3919	1.01 (0.97, 1.20)
Presence of under 5-year-old child	No	149	5546	1.0	689	7089	1.0	546	7563	1.0
	Yes	260	7756	1.25 (0.93, 1.68)	679	8231	0.85 (0.69, 1.04)*	535	8006	0.94 (0.78, 1.13)
Interruption of water sources at least a day in the last 2 weeks	No	–	–	–	–	–	–	–	3743	1.0
	Yes	–	–	–	–	–	–	–	3479	1.55 (0.48, 0.87)*

*COR* crude odds ratio; *, significantly associated; –, no data for analysis

Table 4 shows multivariable regression analysis on the use of appropriate point-of-use water treatment methods and associated factors. There was no collinearity among predictor variables as the multi-collinearity diagnostics did not show variables with VIF of greater than 10. In all surveys, education status of household head and owning radio had a significant association with the household use of the water treatment methods after controlling other variables. The household head with higher education in 2005, 2011, and 2016 had 5.99 (95% CI = 3.48, 10.33), 3.61 (95% CI = 2.56, 5.07) and 3.43 (95% CI = 2.19, 5.37) times higher odds ratio, respectively, to use appropriate treatment methods as compared to household head who had no education. Households owned a radio in 2005, 2011, and 2016 had 1.58 (95% CI = 1.02, 2.47), 1.50 (95% CI = 1.21, 1.86), and 1.34 (95% CI = 1.03, 1.74) times higher odds ratio, respectively, to use the appropriate water treatment methods as compared to the households who did not own radio. There is sufficient evidence to accept the hypothesis that the reported use of appropriate treatment methods is high in the households of educated household heads and owned a radio in all the three surveys. But, we fail to accept the hypothesis that there is a high reported use of the treatment methods in the households located in urban areas, found in high wealth quintiles, owning a television, dependent on unimproved water sources, and having children with age below 5 years in 2005 EDHS surveys. From the pooled data regression, the odds of treating water with an appropriate water treatment were 2.78 (95% CI = 2.16, 3.57) and 2.18 (1.64, 2.89) times higher in 2011 and 2016, respectively, compared to 2005 DHS survey. Households with primary, secondary, and higher education head had 1.71 (1.44, 2.02), 2.17 (1.73, 2.73), and 3.58 (2.79, 4.59) times higher odds to use appropriate water treatment methods, respectively, as compared to household heads with no education. Moreover, the odds of treating water with appropriate household treatment methods is higher in the households of being in higher and highest wealth quintiles, being living in rural, dependent in unimproved water sources, and owning radio and television (Table 5). In a pooled data analysis, there are sufficient evidences to accept the hypothesis that the reported use of appropriate treatment methods is high in the households of living in rural, educated household heads, found in high wealth quintiles, owned a radio, owned a television, and dependent on unimproved water sources.

**Table 4:** Multivariable regression result on factors associated with the household use of appropriate water treatment methods in DHS of 2005, 2011, and 2016, Ethiopia

Variables		2005 EDHS			2011 EDHS			2016 EDHS		
		User	Non-user	AOR (95% CI)	User	Non-user	AOR (95% CI)	User	Non-user	AOR (95% CI)
Residency	Rural	327	11,414	1.0	914	12,002	1.0	724	12,542	1.0
	Urban	82	1888	0.56 (0.28, 1.13)	454	3318	0.32 (0.18, 0.56)*	357	3027	1.11 (0.71, 1.74)
Education of head	No education	177	9059	1.0	486	9040	1.0	377	8706	1.0
	Primary	134	2809	2.07 (1.45, 2.95)*	493	4832	1.56 (1.23, 1.98)*	380	4647	2.12 (1.47, 3.07)*
	Secondary	60	1154	2.18 (1.32, 3.60)*	143	753	2.33 (1.64, 3.31)*	136	1188	2.05 (1.33, 3.15)*
	Higher	36	250	5.99 (3.48, 10.33)*	240	670	3.61 (2.56, 5.07)*	187	981	3.43 (2.19, 5.37)*
Wealth status	Poorest	50	2704	1.0	111	3097	1.0	165	3037	1.0
	Poorer	65	2773	1.11 (0.62, 1.98)	151	3069	1.35 (0.88, 2.09)	128	3076	1.16 (0.53, 2.52)
	Middle	50	2617	0.80 (0.40, 1.62)	177	2914	1.63 (1.04, 2.56) *	150	2971	1.70 (0.85, 3.42)
	Higher	102	2427	1.50 (0.73, 3.10)	303	2764	2.71 (1.67, 4.39)*	201	2882	1.51 (0.78, 2.92)
	Highest	142	2781	1.46 (0.54, 3.98)	626	3476	5.04 (2.62, 9.69)*	436	3603	1.09 (0.50, 2.38)
Water sources	Improved	156	5370	—	797	8159	1.0	697	10,096	-
	Unimproved	253	7931	—	571	7160	1.75 (1.18, 2.60)*	384	5473	-
Had radio	No	188	8901	1.0	522	9395	1.0	658	11,293	1.0
	Yes	221	4396	1.58 (1.02, 2.47)*	846	5922	1.50 (1.21, 1.86)*	423	4276	1.34 (1.03, 1.74)*
Had television	No	368	12,671	1.0	995	13,952	1.0	762	13,592	1.0
	Yes	41	627	1.17 (0.73, 1.98)	371	1359	2.77 (1.93, 3.97)*	319	1977	2.18 (1.33, 3.58)*
Sex of house head	Female	103	3014	—	980	11,355	1.0	776	11,650	1.0
	Male	306	10,288	—	388	3965	1.43 (1.16, 1.76)*	305	3919	1.25 (0.97, 1.59)
Presence of under 5-year-old child	No	149	5546	1.0	689	7089	1.0	546	7563	—
	Yes	260	7756	1.18 (0.86, 1.64)	679	8231	1.01 (0.83, 1.204)	535	8006	—
Interruption of water sources at least a day in the last 2 weeks	No	‾	‾	‾	‾	‾	‾	‾	3743	1.0
	Yes	‾	‾	‾	‾	‾	‾	‾	3479	1.10 (0.79, 1.55)

*AOR* adjusted odds ratio; *, significantly associated; —, excluded from multivariable regression since *p* > 0.25; ‾, no data for analysis; -, removed due to collinearity

**Table 5:** Multivariable regression result of a pooled data of three DHS on factors associated with the household use of appropriate water treatment, Ethiopia

Variables		Appropriate treatment use, weighted frequency		AOR (95% CI)
		User	Non-user	
Survey year	2005	409	13,302	1.0
	2011	1368	15,319	2.78 (2.16, 3.57)*
	2016	1081	15,569	2.18 (1.64, 3.89)*
Residency	Rural	1965	35,957	1.0
	Urban	893	8233	0.49 (0.33, 0.72)*
Education of head	No education	1040	26,805	1.0
	Primary	1006	12,289	1.71 (1.44, 2.02)*
	Secondary	339	3094	2.17 (1.73, 2.73)*
	Higher	463	1901	3.56 (2.77, 4.58)*
Wealth status	Poorest	326	8839	1.0
	Poorer	343	8918	1.03 (0.72, 1.46)
	Middle	377	8502	1.16 (0.77, 1.75)
	Higher	607	8072	1.78 (1.18, 2.67)*
	Highest	1204	9860	2.47 (1.53, 4.00)*
Water sources	Improved	1650	23,626	1.0
	Unimproved	1208	20,565	1.76 (1.39, 2.22)*
Had radio	No	1369	29,590	1.0
	Yes	1489	14,593	1.41 (1.22, 1.62)*
Had television	No	2126	40,214	1.0
	Yes	731	3963	2.25 (1.72, 2.95)*
Sex of house head	Female	796	10,898	1.0
	Male	2063	33,293	1.28 (1.28, 1.68)*
Presence of under 5-year-old child	No	1384	20,197	1.0
	Yes	1474	23,993	1.01 (0.89, 1.16)

*AOR* adjusted odds ratio; *, significantly associated

### Results of multilevel modeling

The data had a two-level hierarchical structure with households at level 1 nested within 11 regions at level 2. We began by the empty (intercept only) two-level model for a dichotomous outcome variable (household use or non-use appropriate treatment methods). The output shows that there was a variation in the use of appropriate water treatment due to the variation in regions. The interclass correlation from empty model indicates that 15.3% of variations in 2005, 9.1% of the variation in 2011, and 29.6% of variations in 2016 are attributable to the difference in the region (Table 6). The variations in the use of treatment methods due to the difference in the region were significant in each survey regardless of whether there was a higher within-region variation compared to between-regions variation. The higher within-region variation than between-regions variations suggests that the use of point-of-use water treatment methods is a nationwide problem.

**Table 6:** Intercept only model on household use of appropriate water treatment methods DHS 2005, 2011, and 2016, Ethiopia

Use of treatment	Empty model (95% CI)		
	2005	2011	2016
Level 1 (the odds of treatment use in an average regions)	0.02 (0.01, 0.03)	0.11 (0.08, 0.15)	0.23 (0.20, 0.26)
Level 2 (the variance of random factor or odds of treatment use between regions)	0.59 (0.24, 1.49)	0.33 (0.14, 0.77)	1.13 (0.56, 3.43)
Interclass correlation in percent	15.3 (6.70, 31.13)	9.1 (4.06, 19.06)	29.6 (14.5, 51.03)

The data were not weighted

Similar to the output of multivariable regression analysis, the fixed part of multilevel modeling indicates that household use of appropriate water treatment options generally was associated with the educational status of household head, wealth status, and ownership of radio and television. The 2016 survey shows that supplying water intermittently to household had 1.26 (95% CI = 1.06, 1.49) times higher odds of using treatment methods regardless of the region where the households are located (result not indicated in the table). In 2005 and 2011 EDHS, households dependent on unimproved water sources were 34% and 37% more likely to use water treatment methods, respectively, compared to households using improved water sources. In 2016, households’ use of either boiling, filtration, bleach, and SODIS was associated with the presence of under 5-year-old children in the household (AOR 1.12; 95% CI = 0.99, 1.27) (Table 7).

**Table 7:** Full multilevel model on household use of appropriate water treatment methods in DHS 2005, 2011, and 2016, Ethiopia

	Variables		OR (95% CI)		
			2005	2011	2016
Fixed part	Residency	Rural	1	1	1
		Urban	0.86 (0.56, 1.33)	0.56 (0.46, 0.68)*	0.90 (0.70, 1.16)
	Education of head	No education	1	1	1
		Primary	1.67 (1.24, 2.26)*	1.47 (1.28, 1.69)*	1.84 (1.58, 2.15)*
		Secondary	2.32 (1.64, 3.26)*	2.19 (1.80, 2.65)*	2.15 (1.76, 2.62)*
		Higher	5.51 (3.63, 8.35)*	2.96 (2.45, 3.57)*	3.01 (2.47, 3.66)*
	Wealth status	Poorest	1	1	1
		Poorer	1.36 (0.82, 2.25)	1.62 (1.26, 2.09)*	0.90 (0.69, 1.16)
		Middle	1.47 (0.88, 2.46)	1.85 (1.43, 2.39)*	1.16 (0.90, 1.50)
		Higher	2.24 (1.37, 3.68)*	2.99 (2.36, 3.78) *	1.49 (1.16, 1.90)*
		Highest	2.447 (1.41, 4.34)*	5.24 (3.99, 6.88) *	1.55 (1.14, 2.11)*
	Water sources	Unimproved	1	1	
		Improved	0.66 (0.48, 0.91)*	0.63 (0.54, 0.72) *	0.51 (0.44, 0.60)*
	Radio ownership	No	1	1	1
		Yes	1.39 (1.03, 1.87)*	1.48 (1.31, 1.67) *	1.21 (1.07, 1.38)*
	Television ownership	No	1	1	1
		Yes	1.52 (1.08, 2.15)*	1.87 (1.59, 2.19) *	1.89 (1.55, 2.30)*
	Sex of house head	Female	1	1	1
		Male	1.50 (1.17, 1.93)*	1.30 (1.15, 1.47) *	1.24 (1.09, 1.41)*
	Presence of under 5-year-old child	No	1	1	1
		Yes	1.02 (0.81, 1.28)	1.06 (0.95, 1.19)	1.07 (0.95, 1.20)*
Random part (region-level variation)			0.39 (0.14, 1.07)	0.17 (0.07, 0.41)	0.11 (0.04, 0.27)

The data used for multilevel modeling were not weighted data, The result of multilevel modeling with the inclusion of intermittent water supply was run independently since it was collinear with water sources; *OR* odds ratio; *, significantly associated

## Discussion

Primary prevention of the diseases consists of manipulation of human environment that includes water supply [15]. Treating water prior to drinking, which can be taken as a primary prevention method for diarrhea, is found to be effective in reducing the disease [4–6]. In Ethiopia, despite having preceding studies that show the effectiveness of point-of-use water treatment in reducing diarrhea [16, 17], it is still among the common health problems [18–20]. Reducing the problem using household water treatment can be ensured when the treatment methods are adopted and effectively and consistently used [21, 22]. To ensure the wide-scale and effective use of household water treatment methods, identifying and intervening the factors affecting the use is indispensable. The current study focused on the household reported use of appropriate household water treatment methods and the factors associated with the use for future intervention.

The analysis of the three EDHS data indicates that there is an increasing trend in the number of households that used water from improved sources. At the same time, the number of households treating water with different point-of-use water treatment methods has increased from 2005 to 2016. Particular to appropriate water treatment use, 3.0%, 8.2%, and 6.5% of households had reportedly used the treatment options respectively in 2005, 2011, and 2016 EDHS.

Household point-of-use water treatment was associated with ownership of radio and television that might be because of conveying of information about the health benefits of treating water and health risk of untreated water by these media. In this regard, a study finding shows that conveying relevant information about household water quality improves the adoption of protective behaviors and technologies [23].

Households in higher and highest wealth quintile had higher odds of using either of the treatment options in the 2005 and 2011 surveys. Similar to our finding, an assessment in Egypt shows that households in the wealthiest quintile were 18.2 times more likely to use filters than those in the remaining four wealth quintiles [24].

The result also indicates that households dependent on unimproved water sources were more likely to use either of the treatment options in the survey of 2011. Our finding complies with a study finding in Zambia that shows that households obtaining water from unimproved sources (rivers and streams) were more likely to chlorinate their water [25]. Another study finding shows that households that considered public water was unsafe for drinking preferred to boil their drinking water prior to consumption [26]. On the other hand, the finding on households dependent on improved water sources was less likely to use treatment methods might be from the households’ perception that improved water is free of pathogens, and post-collection contamination is less likely even if further study is needed in this regard. But, it is arguable that the treatment methods should not only be used by households dependent on unimproved water sources in the country due to the prior report that shows that about 32% of improved water sources do not meet the national standards of microbial load per 100 ml [27].

Moreover, the 2016 survey analysis on households with an intermittent water supply had higher odds of treating their water at the household level is corroborated by a study finding in Egypt that shows households with an intermittent supply were more likely to let the water stand and settle [24].

Household head with at least primary education level had higher odds of treating their water prior to drinking than households heads who had no formal education in each of the three surveys. Our finding is similar to a study finding in Zambia that indicates that chlorine use was more likely among those with post-secondary education [25].

The number of households treating water at point-of-use in 2005 was significantly lower than the two latter surveys. There was an increasing trend from 2005 to 2011 in the number of households using appropriate water treatment methods. The rise in the number of households reportedly using the treatment methods from 2005 to 2011 might be from the emphasis given to demand creation service during this survey time and its persuasiveness. There was a decreasing trend from 2011 to 2016 in the number of households reportedly treating their water decreased. The number of households dependent on improved water sources increased from 2011 to 2016. Therefore, decreasing in the number of households using point-of-use water treatment from 2011 to 2016 might be from the perception of the households to treat improved water sources, less emphasis might be given to promotion services and shortage in the supply of the treatment methods. The declining number of users from 2011 to 2016 also shows limitations in the government effort to scale-up of different treatment methods and disagree with the prior reports [10, 28]. Moreover, it is suspicious that the report on the cooperation of concerned government offices with different organizations that pledge support in facilitating partnerships and effective implementation of HWTS [10].

In the country, since 2003, health extension workers have been deployed and implementing water safety measures including the household water treatment and safe storage practices [29]. The current finding that shows more than 90% of households that do not use any of point-of-use water treatment methods suggests the existence of either a little emphasis or gap in the implementation of the package. In addition, a decreasing trend from 2011 to 2016 contradicts the national plan targeted to reach 35% coverage of household water treatment use and safe storage practices at the end of 2020 [11].

### Strengths and limitations

The EDHS data we analyzed were collected cross-sectionally and, therefore, have the following limitations: (1) The responses were liable to biases (social desirability bias); (2) The analysis fails to show the cause and effect relationship between independent variables and dependent variable [30]. The data did not indicate whether households claimed using the treatment methods were confirmed users and how consistent is the water treatment. In addition, the data did not explicitly indicate different types of filtration methods used by households. Psychosocial factors, one of the factors associated with WASH technology adoption and use, were not included in the data for analysis. The Ethiopia Demographic and Health Survey is conducted in every 5 years via appropriate procedures of selecting households that would represent the whole population in the country. Therefore, the representativeness of the data is one of the strengths when compared with area-specific studies being conducted in the country.

## Conclusions

Below 10% of households had treated their water at point-of-use via appropriated treatment methods during all the three Ethiopian Demographic and Health Surveys. Household and individual level characteristics mainly education status of household head, owning a radio and television, and wealth quintiles had an association with the household use of the treatment methods. Community-level factors mainly being in urban or rural had also a significant association with treatment use in the 2011 survey. There were within-region and between-region variations in the use of treatment methods in each survey. The finding in general suggests the need for designing intervention and implementation strategies at the national level for wide-scale use of the treatment methods which ultimately ensure the health gains. A study on the consistency in the use of the treatment methods among reported users and its effectiveness against diarrheal disease needs to be conducted in the country. Moreover, further study about household behavioral factors related to the safe water system is needed to design appropriate behavior changing intervention strategies.

## Abbreviations

AOR: Adjusted odds ratio
COR: Crude odds ratio
DHS: Demographic and Health Survey
EAs: Enumeration areas
EDHS: Ethiopian Demographic Health Survey
FMOH: Federal Ministry of Health
HWTS: Household water treatment system
ICF: International Classification of Functioning
MoWIE: Ministry of Water, Irrigation and Electricity
SNNPR: Southern Nation, Nationalities and People
SODIS: Solar disinfection
WASH: Water, sanitation, and hygiene
